# Supplementary material for: Prevalence of delayed treatment for sexually transmitted infections and its determinants in sub-Saharan Africa. A systematic review and meta-analysis
Source: PLoS One. 2024 Mar 21;19(3):e0299629. doi: 10.1371/journal.pone.0299629 (PMC10956779; doi:10.1371/journal.pone.0299629)
Supplement: S3 File — (DOCX) [file pone.0299629.s005.docx]

**Supporting file-3: effect of poor knowledge on delayed treatment for STI in sub-Saharan African countries**
