# Supplementary material for: Prevalence of delayed treatment for sexually transmitted infections and its determinants in sub-Saharan Africa. A systematic review and meta-analysis
Source: PLoS One. 2024 Mar 21;19(3):e0299629. doi: 10.1371/journal.pone.0299629 (PMC10956779; doi:10.1371/journal.pone.0299629)
Supplement: Attachment — Submitted filename: respose to reviewers.pdf [file pone.0299629.s008.pdf]

Prevalence of delayed treatment for sexually transmitted infections and its determinants in Sub-Saharan Africa. A systematic review and Meta-analysis

Muluken Chanie Agimas*[1], Milkias Solomon[1], Daniel Alayu Shewaye[1], Dessie Abebaw[1], Nebiyu Mekonnen Derseh[1]

1. College of Medicine and health science, institute of public health, Department of Epidemiology and Biostatistics, University of Gondar, Gondar, Ethiopia.

Authors address:

Muluken Chanie Agimas (MCA): mulukensrc12@gmail.com

Daniel Alayu Shewaye (DAS): danielalayu14@gmail.com

Milkias Solomon (MS): milkiyassolo39@gmail.com

Dessie Abebaw (DA): dessieabebaw96@gmail.com

Nebiyu Mekonnen Derseh (NMD): nebiyumek12@gmail.com

Corresponding Author

MCA: mulukensrc12@gmail.com

To: PLOS ONE <em@editorialmanager.com>

Manuscript Number: PONE-D-23-13983

Cover Letter for reviewer's comments and authors responses

We would like to thank both the Editorial board members and reviewers for kind comments and suggestions. Accordingly, we have tried to correct and respond to those comments point by point, page by page, and line by line. Therefore, we prepared this author's response, the revised manuscript, the separate revised figures and tables, and the tracked changes of the manuscript.

Sincerely, with best regards!

Muluken Chanie Agimas

ID: https://orcid.org/0000-0003-0808-124X

Comments from review and editorial board	Authors response
**Editor's comment**	
Please ensure that your manuscript meets PLOS ONE's style requirements, including those for file naming. The PLOS ONE style templates can be found at	Dear Editor, it is a very interesting comment, and we accepted it so that we can assure you that our manuscript fulfils all the requirements.
Please include your tables as part of your main manuscript and remove the individual files. Please note that supplementary tables (should remain/ be uploaded) as separate "supporting information" files. We note that your Data Availability Statement is currently as follows: [All relevant data are within the manuscript and its Supporting Information files.]Please confirm at this time whether or not your submission contains all raw data required to replicate the results of your study.	Thank you, dear editor. You are right, and we did it.
Please include a separate caption for each figure in your manuscript.	It is great, and you are right. As I read, PLOS ONE requires a separate caption in the manuscript. Therefore, the necessary amendment has been made accordingly.
Please include captions for your Supporting Information files at the end of your manuscript, and update any in-text citations to match accordingly	Thank you so much for your comment. You are right PLOSE ONE recommends the supporting information at the end of manuscript. Therefore we accept and revise it.
**Reviewer-1 comments**	
**ABSTRACT:**<br><br>**-ABSTRACT**, result: a bit confused as no reference group was mentioned (from result in main text it's above secondary school). Also, the terms for "no education", "primary education", "and secondary education" should be revised. Methods of ABSTRACT did not mention how the OR was calculated.	Dear reviewer, It is a very interesting comment. We write this to avoid detailed narrations in the abstract section. You are right; adding what you have asked makes the abstract more informative. The necessary amendment has been made.

**ABSTRACT**, conclusion: risk factors duplicated with results. Suggest to summarize or highlight or just delete it	Thank you so much for your great comments. You are right; it seems redundant. So we accept the comment and revise it.
**INTRDOUCTION:**<br>-The authors are highly recommended to trim the introduction, especially the overlapping parts or sentences. For instance, lines 77-80 about the STI burden could be shortened by selecting either reference source instead of listing both.<br><br>- HIV may not be included as STI as the symptom presentation and reasons for delayed treatment was quite different from common STI. And please double check if HIV was excluded from the literature search. "Bacteria, viruses, and parasites are responsible for STIs such as gonorrhea, chlamydial infection, syphilis, trichomoniasis, chancroid, genital herpes, human immunodeficiency virus infection, and hepatitis B infection."<br><br>-"Among all STIs, syphilis, gonorrhea, chlamydia, and trichomoniasis are curable (8)" They are not the only ones, but the bacterial STI curable and with higher disease burden. So this sentence is misleading with "all" included.<br>-"Thus, the transmission rate of the human immunodeficiency virus … " don't understand this sentence. Do the authors mean STI was a risk factor for HIV infection?	We really appreciate your detailed view. We have seen our manuscripts again, one by one. We especially observed that the introduction needs extensive revision. We accept all the comments you gave on the introduction. Redundant and ambiguous sentences have been removed and revised.<br><br>Regarding "thus, the transmission rate of the human immunodeficiency virus," we mean that the STIs increase the risk of human immunodeficiency virus transmission. We also assure you that HIV was excluded from the literature search.
METHODS:<br><br>-instead of the time period for performing systematic review, it's more important for readers to know the time coverage of literature published. Did the literature search started from very beginning (say 1980) up till March 2023?	Thank you, dear reviewer, for your genuine comment. The reason why we state the time period for searching for literature is to make it clear to the reader the number of articles available during

-please suggest if the eligibility criteria included study design. For calculation of pooled delayed treatment proportion, should RCT be excluded?<br><br>-outcome of the study: the 'time taken" for defining delayed treatment was not clear. So how long of interval was defined as "yes"? actually, I believe the definition of delayed treatment varied across studies which the authors could not control. If that's true, please add a paragraph in RESULTS to summarize the definition of delayed treatment for STI used in studies. This would be useful reference for future studies.<br><br>-has the systematic review registered? Please mention this. If no, please contact editor on how to handle this.	that period. Sometimes, new articles may be published after our searching.<br>For example, if we search articles from March 20 to April 20/2023, articles published after April 20 may not be considered. Someone may ask about missed articles that were published after the search period. In this case, narrating the searching period clarifies these issues. We believe that authors are responsible for missed articles within the search period. The time scope of our search depends on how many articles are available during the search period. That is why we include the searching period.<br>Regarding "time coverage of literature published," We consider all published and unpublished articles available during the searching period. If we do not include all articles at any period of publication/study period, the number of articles for analysis will be very small, which makes it difficult for subgroup analysis and other forms of analysis.<br><br>Therefore, we include both the searching period of the articles and the article publishing year (time coverage of literature published) to be more informative for the reader.

	Dear reviewer, Regarding the definition of delayed STI treatment, as you have seen, it was not clearly stated. An amendment has now been made based on your comment.<br><br>Dear reviewer, Initially, we plan to include all types of study designs. Any study that reported the proportion or determinants of delayed STI was planned for analysis. But as far as our searching effort went, we found articles with a cross-sectional study design and used them for analysis.<br><br>Regarding the protocol registration, there is no systematic or meta-analysis of delayed treatment of STI in sub-Saharan Africa. Additionally, there is no protocol registered or published previously about delayed treatment of STIs in the PROSPERO. Dear reviewer, honestly speaking, we believe that registering in the PROSPERO is optional. Definitely, you are right; registering the protocol in PROSPERO is the first task of systematic review and meta-analysis. We take your comment as a lesson for future work. Otherwise, we will communicate with the editor.

RESULTS:<br><br>-‘handling heterogeneity’: please cite the table/figure for this. Also, what’re the results for “Sensitivity analysis, and subgroup analysis were performed to assess the heterogeneity” ? Without results, the sentence should belong to METHODS.	Dear reviewer, definitely without a result; it is a method. To handle the heterogeneity, we used subgroup analysis by year and region or country. For this output, we cited its corresponding figure (output). So the subheading or title “heterogeneity management” infers about subgroup analysis by year and region. To avoid this ambiguity, we amend it accordingly.
-subgroup analysis by year should use study year instead of publication year. A study could be conducted before 2010 but the publication year was after 2010.	Dear reviewer Thank you so much for your interesting comments. You are right that subgroup analysis by publication year and study period has different output and value. Subgroup analysis by study period gives more meaningful information than publication year. Therefore, amendments have been made accordingly.
-Table 2 should add columns of list of STI included in study, the definition of delayed treatment, recruitment site (e.g. STI treatment clinics, NGOs, online platform) and/or participants (e.g. heterosexual male, heterosexual female, MSM, FSW, clients of FSW, PWID).	Dear reviewer, honestly speaking, articles were not reported about the specific STI type. Instead, articles were conducted on the treatment seeking (delay or early) for any type of STI without specification. But the definition of delayed treatment, recruitment site (study setting), and type of participants (commercial sex workers, truck drivers, adolescents, men, and all age groups)

	were reported. So we can add this information to our article to convey the full information to the reader.
-if possible, authors are recommended to perform subgroup analysis by type of STI, and type of recruitment sites.	Dear reviewer, we faced a challenge to get information about types of STI in the articles. Therefore, we conduct subgroup analysis by recruitment site (study setting).
**DISCUSSION**:<br>-The interpretation of subgroup analysis by year may need caution with some notes. Comparison of studies by year should be, at least, based on the same list of STI. So that we could understand if there was actual increase of delayed treatment over time. Please revise this sentence: "Delayed treatment for STIs was higher after 2010 than before 2010. This means the distribution of delayed treatment for STIs has increased over time in Sub-Saharan African countries."	Dear reviewer, as we mentioned earlier, the previous studies were not conducted on the specific types of STI. It was conducted on treatment-seeking behavior for the overall STI. We cannot find information on the specific type of STI in the selected articles for subgroup analysis.<br><br>Dear reviewer regarding the interpretation, definitely, you are right; the sentence was somewhat unclear. So amendments have been made as follows: So "the pooled prevalence of delayed treatment for STIs was higher among studies conducted after 2010 than before 2010. This may be because the COVID-19 pandemic negatively affects the screening practice for hypertension."

What is “Nework 30%”?	We mean the delayed treatment of STI in New York City was 30%. Sorry for wrongly spelled word.
don’t understand “That means a study in Vietnam was conducted only among women and women who experienced less severe symptoms than men and thus may delay seeking treatment (53).” The “lower” proportion in the previous sentence did not match with this.	Dear reviewer, as you know, women had fewer severe symptoms of STIs than men (which is evidenced by different articles and guidelines). If the symptoms are less severe, they may give less attention to getting treatment promptly. In the study of Vietnam, the study participants were only women. Because of this, they may delay getting the treatment, and overestimate the finding. This was supported by the previous study.
-duplication on the meaning for “Thus, the perception of people about STIs influences treatment-seeking behavior. This means participants perceiving STIs as not serious were at higher risk of delayed treatment for STIs in Sub-Saharan Africa.”	Great! You are right. It was duplicated or unnecessarily detailed, and thank you for the chance to amend the sentence. So we correct it accordingly.
-line 262, “In the qualitative synthesis or systematic review …” should it be meta-analysis instead of systematic review?	Dear reviewer, we used systematic review instead of meta-analysis for the factor not to be pooled. This factor was only reported in one article. In this case, it is preferable to explain or narrate instead of using a pooled estimate. By definition, we used meta-analysis for two or more articles pooled effect, articles. Otherwise, we use systematic review.

Overall: please double check the whole manuscript carefully to make sure there're no duplication of meaning in sentences nearby, no "strange" terms used (e.g. "end or advance into a sexually transmitted diseases", which should be seroclearance or progression …, goals of the sustainable development goal [duplicated]). Please make sure the sentence describing STI is correct and precise in INTRODUCTION.<br><br>Ethical declaration: please check with editor on the suggested sentence for systematic review. "Not practical" is unlikely to be a suitable term.	Thank you, dear reviewer, for your very interesting comment and your scientific contribution to make our work more impressive. We look over our work to make an amendment based on your suggestions.<br><br>Regarding the ethical declaration, we used the published articles for further analysis. Our participants were the articles. Because of this, consent was not obtained from the participants. Otherwise, we will deal with our editor.
**Reviewer two comments**	**Authors response**
In line 68 - 70, consider rephrasing the sentences and avoid starting the paragraph with "Especially". The following sentence "These youth and adolescents are the productive segment of the population and thus the economic fate of a country depends on the youth and adolescent age group a population" requires rephrasing to make sense	Dear reviewer It is a very interesting comment. You have seen our manuscript attentively, and we would like to thank you for your scientific contribution to making our manuscript valuable for intervention and policymaking. We accept your valuable comments. So the necessary amendment has been made accordingly.
In line 72 – 73, can the authors state all and accurate reasons why STIs are common in developing countries which should include management of STIs which is mostly syndrome approaches.	Great, you are right. Such information provides important information for the scientific community. So amendment has been made.
In line 74 – 76, the argument is not correct as most of the STIs are asymptomatic, and patients don't know that they have an infection. I	Great view! We made a mistake. As you stated, the possible factors for the timing of seeking the health service were not

encourage the authors to present factual points on why treatment of STIs is not early.	clearly stated. Now, by taking your comment as a lesson, we have made corrections. Thank you very much for the new insights and knowledge. Really, we are lucky when you are assigned as a reviewer for our paper. We have learned a lot and gained experience from your comments, even for future manuscript preparation.
In line 80 – 81, the authors referenced a figure which I could not review.	Dear reviewer, Normally, we use the appropriate figure. If it is necessary, we will revise the global and sub-Saharan African magnitude of STIs using the recent facts.
In line 98, not all STIs can be cured, the sentence is inaccurate.	Dear reviewer, we accept it and necessary amendment has been made accordingly.
The introduction does not clearly present the background on this topic to show why there is gap on timing of treatment for STIs. Also, it's not clear if authors are focusing on any STI, curable and incurable. Considerable work might need to be done to have a well written and factual introduction.	Dear reviewer, This comment is not only yours but the first reviewer's too. You are right; we feel that the introduction needs revision. So our introduction has been revised by incorporating the following important issues: definition, transmission, distribution of the problem, previous strategies, magnitude, complications, factors or causes, gaps, and the reason why we were motivated to conduct this study.
For statistical analysis, may the authors state how the meta-analysis was done to obtain a pooled prevalence (proportion). Were the data	Dear reviewer Definitely, you are right. The issues you have mentioned are very

transformed in any way? What assumptions were made, e.g. about the distribution of values? There are some considerations, e.g. very small proportions for prevalence of delayed treatment, which can present problems with the logit transformation using inverse variance methods (e.g. Barendregt JJ, et al. J Epidemiol Commun Health 2013;67:975-78), or possible unreliability with some transformations under certain conditions (e.g. Schwarzer G, et al. Res Synth Methods 2019;10:476	important. But we did not report everything we had done to prepare the most short and precise manuscript. Any ways we revise based on your comment. But frankly speaking, we did not work on data transformation using the inverse variance method. Because we fear loss of data nuance and the potential for errors.
Statistical heterogeneity – the authors need to pay much more attention to the extreme heterogeneity in how to investigate it in the methods, to reporting it in the results and to interpretation of these average values in the Discussion. The I-squared value is given in forest plots and is mostly close to 100%. However, there is no mention of this in the Discussion. Prediction intervals, based on tau-squared, might be an additional way of displaying the between study variability, although with such extreme heterogeneity, these are also difficult to interpret.	Dear reviewers, to manage the heterogeneity between the studies, we try to conduct the subgroup analysis by study period, recruitment site of the participants, and region. Additionally, we try to exclude articles to manage heterogeneity. A random effect model was also used to overcome the problem. Generally, we do everything for heterogeneity management. But it was hard to overcome it. Finally, we saw related articles, including articles published in PLOSE One, about how they report the heterogeneity among the studies. These articles reported and interpreted the pooled effect even though there was substantial heterogeneity between the studies. Therefore, we prefer to honestly report the finding of heterogeneity. But it lacked careful interpretation with its I^2 value in the discussion part. Based on

	your comment, we have now interpreted the finding carefully based on what the software provides (I^2 and its p-value), like this: Despite considerable heterogeneity (I^2 = 98.42%, P =<0.001), the pooled prevalence of delayed treatment for STIs was higher among studies conducted after 2010 than before 2010, which was 50% (95% CI: 42%–59%). This may be because the COVID-19 pandemic negatively affects the screening practice for hypertension. Despite considerable heterogeneity (I^2= 98.42%, P =<0.001), this study also revealed that the pooled prevalence of delayed treatment for STI in Sub-Saharan Africa was 47% (95% CI: 42%–51%).

Thank you, dear editor and reviewers, for your great scientific contribution!
