# Supplementary material for: Prevalence of delayed treatment for sexually transmitted infections and its determinants in sub-Saharan Africa. A systematic review and meta-analysis
Source: PLoS One. 2024 Mar 21;19(3):e0299629. doi: 10.1371/journal.pone.0299629 (PMC10956779; doi:10.1371/journal.pone.0299629)
Supplement: Attachment — Submitted filename: second round respose to reviewers.pdf [file pone.0299629.s009.pdf]

Prevalence of delayed treatment for sexually transmitted infections and its determinants in Sub-Saharan Africa. A systematic review and Meta-analysis

Muluken Chanie Agimas*[1], Milkias Solomon[1], Daniel Alayu Shewaye[1], Dessie Abebaw[1], Nebiyu Mekonnen Derseh[1]

1. College of Medicine and health science, institute of public health, Department of Epidemiology and Biostatistics, University of Gondar, Gondar, Ethiopia.

Authors address:

Muluken Chanie Agimas (MCA): mulukensrc12@gmail.com

Daniel Alayu Shewaye (DAS): danielalayu14@gmail.com

Milkias Solomon (MS): milkiyassolo39@gmail.com

Dessie Abebaw (DA): dessieabebaw96@gmail.com

Nebiyu Mekonnen Derseh (NMD): nebiyumek12@gmail.com

Corresponding Author

MCA: mulukensrc12@gmail.com

To: PLOS ONE <em@editorialmanager.com>

Manuscript Number: PONE-D-23-13983

Cover Letter for reviewer's second round comments and authors responses

We would like to thank both the Editorial board members and reviewers for kind comments and suggestions. Accordingly, we have tried to correct and respond to those comments point by point, page by page, and line by line. Therefore, we prepared this second round author's response, the revised manuscript, the separate revised figures and tables, and the tracked changes of the manuscript.

Sincerely, with best regards!

Muluken Chanie Agimas

ID: https://orcid.org/0000-0003-0808-124X

Comments from review and editorial board	Authors response
**ABSTRACT**<br><br>Please remove the newly added reference group for binary variables, e.g. "as compared to urban". My previous comment referred to education level variable only, as that included more than 2 categories.	Dear reviewer We wrongly interpreted your comment. Sorry for this. So we accepted and got your comment. A necessary amendment has been made accordingly in this section.
**INRODUCTION**<br>I disagree with 'Because of sexual behavior, the burden of STIs is higher in developing countries.' which could result in misinterpretation of people living in developing countries have more sexual behavior. Please revise or remove this and the sentence that follows.<br><br>In general, please double check the grammar of the whole manuscript after the revision. Some newly revised parts do not fit well with the original sentences.	Thank you, dear reviewer. You are right, the sentence somewhat makes sense, and therefore we have removed this ambiguous sentence. Regarding the grammar of the document, as much as possible, we check the grammar carefully and attentively. Therefore, a necessary amendment has been made to the grammar.<br>Regarding the comment **"Some newly revised parts do not fit well with the original sentences".** Dear reviewer, as you know, the introduction section needs extensive revision. Both you and the second reviewer recommend that we rewrite the introduction section. Based on your comments, the introduction section was rewritten in a smart new way. That is why some newly revised parts do not fit well with the original sentences.

Reviewer two	Response of the authors
There are a number of points which were raised in the first review and not addressed in the revised submission.<br>1. A number of points to address in the introduction were not done.<br>2. In the method section, authors were asked to include the time period of the literature search and its still not defined in under the search strategy.<br>3. Authors were requested to define inclusion criteria (study design, participants/population of interest, etc).	Dear reviewer, we really appreciate your detailed view. We have seen our manuscripts once again, one by one. We especially observed that the introduction needs extensive revision. We accept all the comments you gave on the introduction. Regarding "the types of STIs", we consider and write about any type of STI (both curable and incurable STIs). To be clear, we have explained it in brackets like this: "Self-medicating behavior, physical and financial affordability of health services, poor knowledge about STIs, and low socioeconomic status are the risk factors for the timing of treatment seeking for both curable and incurable STIs". "Unless specify, the word STIs is for any STIs. I hope the reader can understand it. Therefore the word STIs in the introduction implies for **both curable and incurable STIs**. Thus, we try to define the definition of STIs, impact, risk factor, magnitude for any type of STIs.<br>Regarding about **"show why there is gap on timing of treatment for STIs."** we clearly explain it **in line 90-95**.<br>In these lines, we try to state why people delay treating for STIs.

	We hope that the introduction is now logically sequenced and well written.<br>Regarding **“time coverage of literature published,"** We consider all published and unpublished articles available during the searching period. If we do not include all articles at any period of publication or study, the number of articles for analysis will be very small, which makes it difficult for subgroup analysis and other forms of analysis.<br>Therefore, the time coverage of published and unpublished literature included for this study was articles published or studied at any time period (starting from the very beginning until the last searching date).<br>**Regarding inclusion criteria:**<br>Dear reviewer, Initially, we plan to include all cross-sectional studies, case-control study designs, experimental study designs, and cohort study designs. But as far as our searching effort went, we found only articles with a cross-sectional study design and used them for analysis.<br>Therefore the eligibility criteria was revised as follows

	**Eligibility criteria**<br><br>**Inclusion criteria**<br><br>• Those articles that addressed either the proportion of delay in health care seeking or determinants of delay in health care seeking for STIs were included.<br>• Articles conducted by cross-sectional studies, prospective or retrospective cohort studies, experimental studies, and case-control studies were potentially illegible for this systematic review and meta-analysis.<br>• Articles written in English and done in sub-Saharan African countries were included.<br>• All sexually active populations (both male and female) were included.<br><br>**Exclusion criteria**

	• Articles with no outcome of interest or full text were excluded.<br>• Short reports, case series, case reports, letters to the editor, comments, and opinions were excluded.

Thank you, dear editor and reviewers, for your great scientific contribution!
