# Supplementary material for: Prevalence of delayed treatment for sexually transmitted infections and its determinants in sub-Saharan Africa. A systematic review and meta-analysis
Source: PLoS One. 2024 Mar 21;19(3):e0299629. doi: 10.1371/journal.pone.0299629 (PMC10956779; doi:10.1371/journal.pone.0299629)
Supplement: Attachment — Submitted filename: Plos One review.docx [file pone.0299629.s007.docx]

COMMENTS

The authors sought to determine the prevalence of delayed treatment for STIs and its determinants in sub-Saharan Africa. A systematic review and meta-analysis. This is an extensive and ambitious review of an important subject. The authors have looked for published and unpublished studies in several databases of prevalent delayed treatment for STIs in sub-Saharan Africa. I could not clearly consider the expertise of the authors.

This is an important paper. There are parts in the introduction which would need considerable attention before the manuscript can be considered for publication.

1. In line 68 - 70, consider rephrasing the sentences and avoid starting the paragraph with “Especially”. The following sentence “These youth and adolescents are the productive segment of the population and thus the economic fate of a country depends on the youth and adolescent age group a population” requires rephrasing to make sense.
2. In line 72 – 73, can the authors state all and accurate reasons why STIs are common in developing countries which should include management of STIs which is mostly syndromic approaches.
3. In line 74 – 76, the argument is not correct as most of the STIs are asymptomatic, and patients don’t know that they have an infection. I encourage the authors to present factual points on why treatment of STIs is not early.
4. In line 80 – 81, the authors referenced a figure which I could not review.
5. In line 98, not all STIs can be cured, the sentence is inaccurate.
6. The introduction does not clearly present the background on this topic to show why there is gap on timing of treatment for STIs. Also, it’s not clear if authors are focusing on any STI, curable and incurable. Considerable work might need to be done to have a well written and factual introduction.
7. For statistical analysis, may the authors state how the meta-analysis was done to obtain a pooled prevalence (proportion). Were the data transformed in any way? What assumptions were made, e.g. about the distribution of values? There are some considerations, e.g. very small proportions for prevalence of delayed treatment, which can present problems with the logit transformation using inverse variance methods (e.g. Barendregt JJ, et al. J Epidemiol Commun Health 2013;67:975-78), or possible unreliability with some transformations under certain conditions (e.g. Schwarzer G, et al. Res Synth Methods 2019;10:476
8. Statistical heterogeneity – the authors need to pay much more attention to the extreme heterogeneity in how to investigate it in the methods, to reporting it in the results and to interpretation of these average values in the Discussion. The I-squared value is given in forest plots and is mostly close to 100%. However, there is no mention of this in the Discussion. Prediction intervals, based on tau-squared, might be an additional way of displaying the between study variability, although with such extreme heterogeneity, these are also difficult to interpret.
